# The Controversy of Using Insufficient Great Saphenous Veins in Coronary Artery Bypass Grafting: A Comparative Morphological Analysis of Healthy and Insufficient Veins Related to the Possibility of Using Them as a Graft

**DOI:** 10.3390/biomedicines12030476

**Published:** 2024-02-21

**Authors:** Andrei Florin Părău, Andrei Raul Manzur, Stefan Mihaicuta, Ioan Adrian Petrache

**Affiliations:** 1 Abdominal Surgery and Phlebology Research Center, “Victor Babes” University of Medicine and Pharmacy Timisoara, 300041 Timisoara, Romania; parau.andrei@umft.ro; 2 1’st Surgical Department, “Pius Brînzeu” University Clinical Hospital Timișoara, 300723 Timisoara, Romania; 3 Department of Doctoral Studies, “Victor Babes” University of Medicine and Pharmacy Timisoara, Eftimie Murgu Square No. 2, 300041 Timisoara, Romania; 4 Department of Cardiovascular Surgery, Institute for Cardiovascular Diseases, 300391 Timisoara, Romania; 5 Center for Research and Innovation in Precision Medicine of Respiratory Diseases, Department of Pulmonology, “Victor Babes” University of Medicine and Pharmacy Timisoara, Eftimie Murgu Square No. 2, 300041 Timisoara, Romania; stefan.mihaicuta@umft.ro; 6 First Discipline of Surgical Semiology, First Department of Surgery, “Victor Babes” University of Medicine and Pharmacy Timisoara, Eftimie Murgu Square No. 2, 300041 Timisoara, Romania; ioan.petrache@umft.ro; 7 Clinic of Thoracic Surgery, Emergency Clinical Municipal Hospital Timisoara, Gheorghe Dima Street No. 5, 300079 Timisoara, Romania

**Keywords:** varicose veins, vein graft, venous wall morphology, coronary bypass

## Abstract

Background: Despite advancements in coronary artery bypass grafting (CABG), the optimal choice of graft material remains a subject of investigation. This study aimed to comprehensively analyze the morphological characteristics of varicose veins, exploring their potential utilization in CABG compared to healthy veins. Methods: The study included 178 patients, categorized into two groups based on healthy and varicose veins. Morphological parameters, including maximum venous diameter, wall thickness, and specific changes in tunica intima (TI), tunica media (TM), and tunica adventitia (TA), were analyzed through microscopic evaluation. Results: Varicose veins exhibited a significantly larger maximum venous diameter (*p* = 0.0001) and increased wall thickness (*p* = 0.0001) compared to healthy veins. Although varicose veins showed thickening in TI and TM, the differences were not statistically significant. Notably, disorganized smooth muscle bundles were more prevalent in varicose veins (*p* = 0.001), suggesting potential wall weakness. The absence of vasa vasorum in TA was significantly higher in varicose veins (*p* = 0.050), influencing vascularization considerations. Conclusions: The comparative morphological microscopic analysis of the specimens of healthy and varicose veins reveals significant differences between the groups, which make the conclusion of this study to plead for avoiding the use of varicose veins as a graft.

## 1. Introduction

Major progress in the diagnosis and in the treatment of acute coronary syndromes has been made in the last decades, and a decrease in mortality has been reported in several countries [1]. However, acute coronary syndromes still are a frequent pathology worldwide. Coronary artery bypass grafting (CABG) remains among the main emergency therapeutic options in case of a severe heart attack, and at the same time, it is electively practiced in many patients suffering from coronary heart disease to reduce the risk of sudden cardiac death [2,3,4].

Surgical coronary bypass can be performed using different graft materials. Autologous greater saphenous vein (GSV) grafting is the treatment of choice because of its superior long-term patency [5]. The use of varicose vein grafts remains a controversial topic of discussion, and few prospective case studies have been reported, with evidence insufficient to draw conclusions at this point [6]. However, the subject divides vascular surgeons into two sides, those who support the use of varicose veins as grafts due to their patency and those who do not.

Varicose veins are not generally used as arterial bypass grafts despite their physiological endothelial flow surface because the large, irregular diameter and the thin wall render these veins inadequate. However, if no other autologous vein is accessible for use as a conduit in lower-limb bypass, varicose vein transplants may be employed. Experimental studies have shown that a considerable reduction in the diameter of veins can be achieved by external wrapping without the generation of obstructing folds of the vein wall. A Dacron mesh tube surrounding the varicose vein can be used [7,8]. Addressing the challenges posed by insufficient GSV may require innovative approaches or mitigation strategies. Techniques such as external wrapping or mesh tube application, as seen in lower-limb bypass procedures, could potentially be explored to enhance the structural integrity of these veins for CABG.

Few reports have delved into the clinical outcomes of lower-limb bypass procedures employing varicose vein grafts, creating a discernible gap in our understanding of their efficacy. Surprisingly, the incidence of major complications associated with varicose vein transplants does not appear to surpass that of conventional saphenous vein grafts. This observation challenges conventional reservations and prompts a reconsideration of the potential utility of varicose veins in bypass surgeries. In the context of coronary and vascular diseases, particularly the high prevalence of chronic venous disease [9,10], the exploration of alternative graft options gains significance. It is noteworthy that varicose veins, despite their historical exclusion from the pool of autologous veins for grafting, may offer a pragmatic solution when suitable alternatives are lacking. This notion is supported by literature emphasizing the associations between venous and coronary diseases [11,12]. In cases where other autologous veins are unavailable, the use of varicose vein grafts emerges as a potential strategy, challenging preconceived notions and necessitating a closer examination of their clinical outcomes.

Moreover, innovative approaches have been proposed, leveraging surgical methods traditionally employed in varicose vein removal for vein-graft harvesting [13]. These methods, adapted from the realm of varicose vein surgery, not only provide a new perspective on graft harvesting but also underscore the adaptability of established surgical techniques to address challenges in coronary artery bypass grafting. This dual-purpose application not only minimizes invasiveness but also enhances the availability of viable graft options for surgeons facing constraints in autologous vein selection. The characteristic association between chronic venous disease and coronary conditions further advocates for the exploration of varicose veins as viable conduits when conventional options are limited. Additionally, the application of varicose vein surgical removal techniques in vein-graft harvesting introduces a novel dimension to graft procurement, showcasing the adaptability of surgical practices in optimizing coronary artery bypass procedures.

To our knowledge, no comprehensive study has microscopically examined comparatively the healthy and varicose veins, in order to establish the possibility to use them as grafts in cardiac or vascular surgery. This paper aims to present a comparative analysis between healthy and varicose venous specimens, in order to establish if the morphology of varicose degenerated veins still allows their use as venous grafts.

## 2. Materials and Methods

### 2.1. Patients and Enrolment Criteria

**Figure 1:**
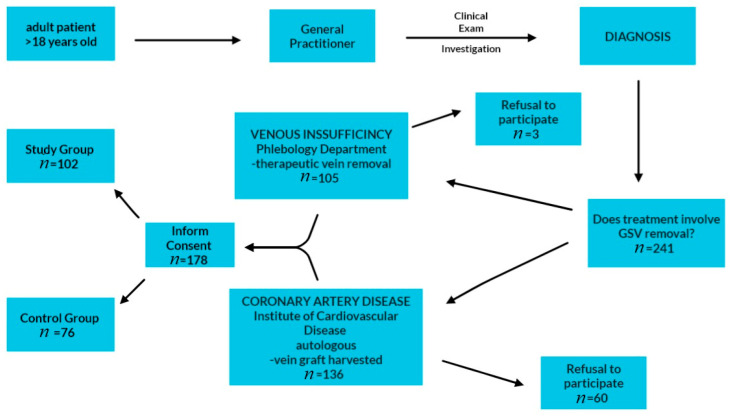
Patient inclusion criteria.

This prospective study included patients evaluated in public health services in Timisoara, Romania, between September 2022 and September 2023, with healthy or varicose lower-limb veins, which underwent different surgeries in which at least one venous specimen was harvested. The patients were first evaluated in the general practitioner’s service and then were sent to the surgery department. All the patients evaluated in the set period of time who signed the informed consent in order to be enrolled were admitted to the study. Of the total number of patients evaluated in the general practitioner’s offices during the established period, 241 required treatments involving GSV removal (therapeutic or to be used as a graft). Sixty-three patients refused to participate in the study. A total number of 178 patients signed the informed consent and were admitted to the study. According to their pathology and surgical indication, 102 patients diagnosed with venous reflux at GSV or GSV collaterals level, were sent to the Phlebology Department, “Pius Brînzeu” University Clinical Hospital Timișoara. They underwent open surgery removal procedures (conventional saphenectomy or phlebectomies). All the venous specimens being harvested—venous specimens from these patients—were included in the study group. Seventy-six patients were diagnosed with coronary artery disease, with more than 75% of vascular lumen occluded. Those were sent to the Institute of Cardiovascular and Heart Diseases, Timișoara, and underwent coronary bypass surgery. The inclusion criteria of the patients are represented in Figure 1. A piece of about 2–5 cm from each healthy great saphenous vein (GSV) used as a graft was harvested for the study—venous specimens from these patients were included in the study’s control group.

### 2.2. Specimen Preparation, Microscopic Evaluation, and Image Analysis

All the specimens were collected in fixative solution (10% buffered formalin) and analyzed by the Pathology Laboratory. For each specimen, 2–3 cross-sections were made in the areas of maximum wall thickness. The processing of the parts was performed manually, by dehydration in alcoholic solutions, clearing, paraffining, and paraffin inclusion, and sections with a thickness of 4 μm were made. For each case, two successive sections were obtained, these being colored in the usual Hematoxylin–Eosin (H&E) stain and the Masson trichrome stain, using the standard recommended protocols. The sections were analyzed on Zeiss Axiocam 506 microscopes (Jena, Germany) and Nikon AY260 (Tokyo, Japan), both equipped with a real-time imaging system and software for digital microscopic image analysis. The examination of the sections displayed on the slides was performed by a team of two pathologists. They both analyzed each specimen, the final result of the interpretation representing their common conclusion.

### 2.3. Statistical Analyses

In this study, the choice of statistical methods was guided by the nature and distribution of our data. For normally distributed data with homogeneity of variance, we utilized the *t*-test, incorporating Welch’s correction as necessary. In cases of non-normally distributed data, we employed nonparametric methods like the Mann–Whitney U Test. It is crucial to note that the unequal group sizes in our study (control group *n* = 76, study group *n* = 102) have implications for the statistical power of our tests. Larger sample sizes typically provide greater power to detect an effect. Consequently, results from the smaller control group should be interpreted with heightened caution, especially in instances of non-significant findings. This consideration is vital in ensuring accurate interpretation and applicability of our results, as it acknowledges the inherent limitations posed by unequal sample sizes in statistical analysis.

## 3. Results

The study compared a control group-healthy veins (*n* = 76) and a study group-varicose veins (*n* = 102) across various parameters, including age, BMI, and morphological characteristics of veins (Table 1). Notably, the mean age in the control group was 63.59 years, while the study group exhibited a significantly lower mean age of 55.68 years. Despite this age difference, BMI did not show a statistically significant distinction between the control and study groups (*p* = 0.59), indicating that both groups were comparable in terms of this parameter.

One of the most noticeable observations was the substantial difference in maximum venous diameter between the control group (mean 0.52 cm) and the study group (mean 0.68 cm), with a highly significant *p*-value of 0.0002. This revealed marked dilatation and tortuosity in varicose veins compared to their healthy counterparts. Wall thickness in the study group (mean 0.206 cm) was significantly higher than in the control group (mean 0.138 cm), with a *p*-value of 0.014. This indicated structural changes and increased vein wall thickness in the study group.

Delving into specific layers of the vascular wall, the thickening of both tunica intima (TI) and tunica media (TM) exhibited statistically significant differences between the groups (*p* = 0.0001 for both). Such thickening is indicative of significant morphological alterations in varicose veins. The study also revealed a noteworthy difference in smooth muscle bundle disorganization between the control and study groups (*p* =0.001), suggesting that varicose veins are more likely to have disorganized smooth muscle cells, potentially contributing to vein wall weakness and dysfunction.

**Figure 2:**
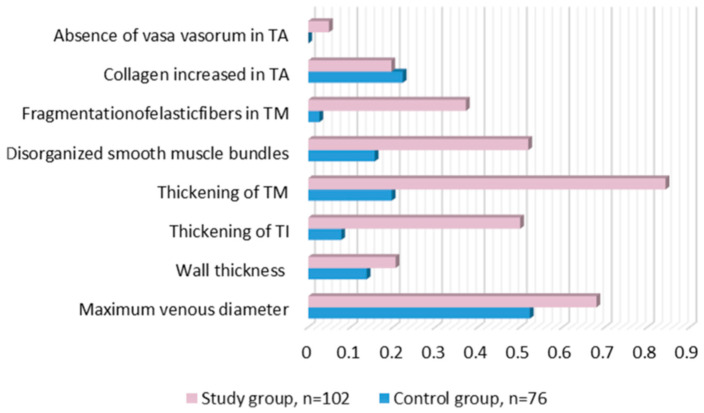
Morphological examination of veins in study and control veins, results according to *p*-value.

Furthermore, the fragmentation of elastic fibers in TM exhibited a significant difference between the two groups (*p* = 0.0001). This finding adds another layer to the structural changes observed in varicose veins, emphasizing the disarray in the organization of key structural components. Although collagen increase in tunica adventitia (TA) showed no significant difference between the control and study groups (*p* = 0.655), the absence of vasa vasorum in TA in the study group (0.0490 ± 0.217) exhibited a significant difference compared to the control group (*p* = 0.050). All these morphological changes are represented in Figure 2.

**Figure 3:**
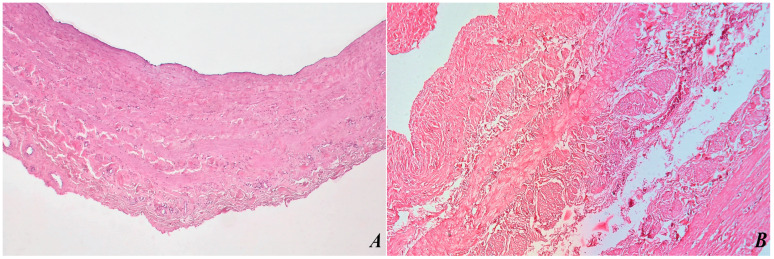
A cross-section of a healthy vein with the three normal layers, original magnification ×100, H&E stain (**A**). A varicose vein, with thickening of TI and TM, fragmentation of elastic fibers, original magnification ×200 (**B**).

**Figure 4:**
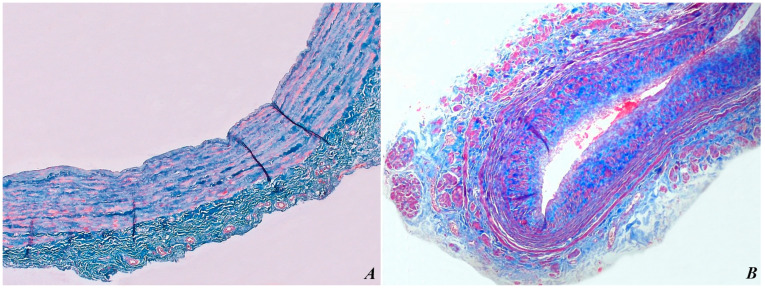
A cross-section of a healthy vein (**A**) and varicose vein (**B**), original magnification ×100, Masson’s trichrome stain. In the varicose vein, the vein wall is thickened, and the TI is disorganized. TM is also thickened and shows signs of disorganization of smooth muscle cells and fragmentation of elastic fibers. TA is also thickened and shows evidence of increased collagen deposition.

The morphological changes identified in the veins were elucidated through microscopic evaluation using Hematoxylin–Eosin (H&E) (Figure 3) and Masson’s trichrome stains (Figure 4). Compared to a healthy vein illustrated in Figure 3A and Figure 4A, Figure 3B and Figure 4B illustrate a varicose vein showing distinct pathological changes. The original magnification of ×200 provides a closer examination, revealing notable deviations from normal vascular architecture. The tunica intima and tunica media of the varicose vein show significant thickening, indicating structural changes in the vessel wall. A closer examination of Figure 4 reveals fragmentation of elastic fibers, a characteristic feature associated with varicose veins. Destruction of elastic fibers compromises the structural integrity of the vein, contributing to the dilation and tortuosity seen in varicose veins.

Higher magnification allows for a more detailed exploration of the morphological changes present in the varicose vein, providing valuable information about the histopathological changes associated with this common vascular condition. Understanding the structural changes underlying varicose veins is essential for developing targeted therapeutic interventions and improving our understanding of venous disorders.

These findings collectively contribute to a nuanced understanding of the morphological disparities between healthy and varicose veins, laying the groundwork for further exploration into the clinical implications of these differences, particularly in the context of CABG.

## 4. Discussion

Coronary artery bypass grafting (CABG) stands at the forefront of cardiovascular interventions, offering various options for graft selection, encompassing both biological and synthetic conduits. Recommendations for bypass vessel selection include an array of possibilities, such as left internal thoracic artery-anterior descending branch anastomosis or grafts harvested from radial, internal thoracic, or gastroepiploic arteries [14]. Despite this diversity, autologous saphenous vein grafts (SVGs) remain the most commonly employed conduits in CABG, especially in cases of multivessel coronary artery disease. This preference is rooted in their established long-term patency and a reduced risk of leg wound complications [14,15,16]. However, the contentious debate persists, with some authors advocating for the use of varicose veins as bypass grafts in specific instances [6,8,17].

There are several possibilities for harvesting venous grafts, including conventional open surgery or an endovascular approach. Using the conventional technique, the saphenous vein is harvested via a large open incision and excised in such a way that causes both vascular damage and wound healing complications [15]. Over the last decade in developed countries, endoscopic vein harvesting has been the method of choice to harvest the greater saphenous vein [18,19]. There are studies that describe a better long-term patency for endoscopic harvested veins [14,18], but analyzing the rate of major adverse cardiovascular events after the bypass operation, the results are similar regardless of the vein harvesting method [19]. Cryostripping can also be used in vein harvesting [13]. All the specimens analyzed in our study were harvested through open procedures in a similar manner. Thus, we do not consider the possibility of thermal or mechanical damage to the analyzed specimens, nor of other injury occurrences which may produce morphology modifications in some way during the harvesting procedure.

The comparative morphological analysis between healthy and insufficient great saphenous veins (GSV) for potential use in coronary artery bypass grafting (CABG) reveals intriguing insights into graft selection for cardiovascular interventions. The observed morphological disparities, particularly in diameter and wall thickness, suggest that insufficient GSVs may pose challenges as grafts in CABG. The significant variations in these parameters could impact the overall effectiveness and durability of the grafts.

Compared with the normal control of the saphenous vein, varicose vein sections showed increased diameter of the lumen and hypertrophy of the wall, mainly of the intima, due to increased amounts of collagen fibers. Collagen fibers also lost their normal pattern and showed abnormal forms. Elastic fibers lost their regular laminar arrangement and formed clumps or scattered fragments [20,21]. In our study in some of the cases, the fibrosis was extended to the entire venous wall. A particular aspect was the diffuse collagenization of the subintimal space, occupied in the cases evaluated with value 3 of score mostly by the presence of an amorphous collagen-type material that greatly modifies the architecture of the venous wall. The accumulation of collagen fibers was correlated with the depletion of reticulin fibers. The collagen fibers partially or completely replace the network in the media and intima. Reticulin fiber depletion reported in the study groups may be considered the result of the long-term effects of varicose disease. 

Microscopically, analysis of varicose veins samples reported in other papers revealed damaged endothelium areas, significant disorganization of the smooth muscle bundles, and the highest density of the vasa vasorum in the tunica media and tunica adventitia, as well as sclerotic blood vessels and neo-angiogenesis in almost all specimens. Immunohistochemistry (IHC) studies showed statistically significant differences between the varicose veins and healthy veins of several parameters, such as protein gene product 9.5-positive structures and laminin-positive structures in the subendothelial layer of varicose veins. There is also the tendency for an increase intra-vascular endothelial growth factor expression and a decrease in collagen type IV structures. Varicose veins represent nonhomogeneous integrity of the basement membrane, smooth muscle disorganization, and active neo-angiogenesis, suggesting remodulation of blood vessels. Changes in the appearance of protein gene product 9.5-containing innervation, laminin, and collagen type IV in tunica intima confirm the remodulation of damaged blood vessels [21].

The importance of assessing the structural integrity of the large saphenous vein insufficiency (GSV) in coronary artery bypass grafting (CABG) cannot be overestimated. Concerns arising from potentially disorganized smooth muscle bundles and attached elastic fibers raise substantial questions regarding the mechanical strength and functional efficacy of these veins as conduits in CABG procedures. While some authors advocate the use of varicose veins as grafts, citing their physiological endothelial flow surface, our meticulous morphological analysis brings to light the profound changes that occur even in the intimal layer of a varicose vein when affected by chronic venous disease (CVD) [7].

The physiological endothelial flow surface, once a compelling argument in favor of varicose veins as grafts, is juxtaposed with our findings revealing modifications within the intimal layer post-CVD affliction. The compromised endothelium, a significant etiological factor for turbulent blood flow, emerges as a critical concern, potentially paving the way for complications, particularly thrombosis [22]. Acknowledging the natural evolution of chronic venous disease and its inflammatory component [23], it becomes imperative to consider the long-term implications. There exists a looming risk of progressive intimal venous hypertrophy, posing a formidable threat to the success of vein grafts in coronary artery bypass grafting [24,25].

In such cases, where the trajectory suggests potential vein graft stenosis or failure, an additional graft stenting procedure becomes a necessity. This intervention aims to mitigate the intimal hyperplasia area and thickness, enhancing lumen uniformity—an aspect assessed with the Fitzgibbon I classification [26]. The meticulous consideration of these factors, grounded in the insights from our morphological analysis, emphasizes the intricate balance between the structural attributes of veins and their long-term functionality as conduits in CABG.

Furthermore, our observations align with existing literature that underscores the multifaceted nature of varicose veins’ structural modifications. The increased diameter of the lumen, hypertrophy of the wall—particularly the intima—due to heightened collagen fiber content, and the irregularities observed in collagen and elastic fibers, all contribute to a complex narrative of varicose vein degeneration. Fibrosis extending to the entire venous wall and the diffuse collagenization of the subintimal space, as noted in our study, corroborate the enduring effects of varicose disease on vein morphology.

Immunohistochemistry plays a pivotal role in elucidating the molecular and cellular characteristics of veins, providing valuable insights into their structural composition and pathological alterations. By utilizing specific antibodies to target proteins of interest, IHC enables the visualization and localization of key molecular markers within vein tissues [27,28]. Commonly employed markers include those indicative of endothelial cells, smooth muscle components, inflammatory cells, and extracellular matrix proteins [29]. This technique aids in understanding the intricate interplay of cellular and molecular factors contributing to vascular health or disease. However, in the scope of the present study, immunohistochemistry was not employed for vein evaluation. The focus of our investigation primarily centered on morphological analysis, utilizing traditional staining methods such as Hematoxylin–Eosin and Masson’s trichrome. The decision to forego immunohistochemistry was driven by the study’s specific objectives, which aimed to comprehensively compare healthy and varicose veins at a morphological level. While IHC undoubtedly offers detailed molecular information, its omission in this study allowed for a streamlined examination of structural changes, paving the way for future research that may delve into the molecular intricacies using immunohistochemical techniques.

The morphological analysis of veins, especially in the context of varicose veins, is crucial for a deeper understanding of vascular pathologies. Our study sheds light on significant differences in the structural integrity and organization of varicose veins compared to healthy veins. These differences, including increased vein diameter, wall thickness, and alterations in smooth muscle and collagen structure, highlight the complex nature of venous remodeling in varicose veins. Understanding these morphological changes is vital for developing more effective treatments and could potentially influence the selection of veins for grafting in coronary artery bypass grafting (CABG). Our findings underscore the necessity for careful consideration of vein morphology in clinical decision-making and pave the way for future research that may explore the functional implications of these structural differences.

It is necessary to discuss some study limitations, too. First of all, the data obtained refer strictly to the morphological comparison of the collected venous specimens and do not provide clinical data regarding their outcomes. The situation resulting from the study is not about clinical validity, being in the experimental stage. Further studies are recommended in order to provide more data and the clinical validity of those results. Secondly, the relatively small number of cases can also be considered a disadvantage. However, this paper lays the groundwork for future longitudinal research to evaluate the long-term performance of insufficient GSV as grafts. Comprehensive clinical trials and patient follow-ups are certainly imperative to assess the success rates, patency, and overall efficacy of CABG by utilizing these veins.

## 5. Conclusions

The comparative morphological microscopic analysis of the specimens of healthy and varicose veins reveals significant differences between the groups, which make the conclusion of this study to plead for avoiding the use of varicose veins as a graft. However, for a final conclusion of the dispute between the categorical use or non-use of varicose veins as grafts, studies on larger groups of patients including the evaluation of the long-term clinical outcomes of these grafts should be indicated.

## Figures and Tables

**Table 1:** Analysis of study parameters and morphological examination of veins (SD = standard deviation; BMI = body mass index; TI = tunica intima; TM = tunica media; TA = tunica adventitia).

Parameter	Control Group, *n* = 76 Mean ± SD	Study Group, *n* = 102 Mean ± SD		*p*-Value
Type of surgery	open saphenous vein harvest *n* = 76	phlebectomies *n* = 7	conventional saphenectomy *n* = 95	
Age	63.592 ± 9.950	55.686 ± 7.900		0.297
BMI	28.616 ± 4.348	28.276 ± 4.169		0.598
Morphological examination of veins				
Maximum venous diameter (cm)	0.523 ± 0.110	0.680 ± 0.314		0.0002
Wall thickness (cm)	0.138 ± 0.092	0.206 ± 0.121		0.014
Thickening of TI	0.078 ± 0.271	0.500 ± 0.502		0.0001
Thickening of TM	0.197 ± 0.400	0.843 ± 0.365		0.0001
Disorganized smooth muscle bundles	0.157 ± 0.367	0.519 ± 0.502		0.005
Fragmentation of elastic fibers in TM	0.0263 ± 0.161	0.372 ± 0.485		0.0001
Collagen increased in TA	0.223 ± 0.419	0.196 ± 0.399		0.655
Absence of vasa vasorum in TA	0.000	0.0490 ± 0.217		0.050

## Data Availability

The data generated in this study may be requested from the corresponding author.
